# Chymase Dependent Pathway of Angiotensin II Generation and Rapeseed Derived Peptides for Antihypertensive Treatment of Spontaneously Hypertensive Rats

**DOI:** 10.3389/fphar.2021.658805

**Published:** 2021-05-17

**Authors:** Iwona Baranowska, Olga Gawrys, Malwina M. Roszkowska-Chojecka, Bozena Badzynska, Dagmara Tymecka, Krzysztof H. Olszynski, Elzbieta Kompanowska-Jezierska

**Affiliations:** ^1^ Department of Renal and Body Fluid Physiology, Mossakowski Medical Research Institute, Polish Academy of Sciences, Warsaw, Poland; ^2^ Faculty of Chemistry, University of Warsaw, Warsaw, Poland; ^3^ Behaviour and Metabolism Research Laboratory, Mossakowski Medical Research Institute, Polish Academy of Sciences, Warsaw, Poland

**Keywords:** chymase, CPPs, rapakinin, RAAS, chymostatin

## Abstract

The contribution of chymase, one of the enzymes responsible for angiotensin II generation in non-ACE pathway, remains unclear in the development of hypertension. The aim of the study was to investigate chymase inhibition as potential antihypertensive therapy in spontaneously hypertensive rats (SHR). To block chymase we employed chymostatin, a commercial inhibitor, and new analogues of rapeseed-derived peptides, VWIS and RIY. These simple and easy to obtain peptides not only block chymase, but also possess weak activity to inhibit ACE. This is a first attempt to evaluate the impact of chronic administration of selected inhibitors on blood pressure of SHR in two phases of hypertension. Male SHR (6 or 16 weeks old) were treated daily for two weeks with chymostatin (CH; 2 mg/kg/day), the peptides VWIS (12.5 mg/kg/day) or RIY (7.5 mg/kg/day); control groups received chymostatin solvent (0.15% DMSO in saline) or peptide solvent (saline). The substances were administered intravenously to conscious animals *via* a chronically cannulated femoral vein. Systolic blood pressure (SBP) was measured by telemetry. Metabolic parameters were measured weekly, and tissue samples were harvested after two weeks of treatment. None of the administered chymase inhibitors affected the development of hypertension in young rats. Only RIY exhibited beneficial properties when administered in the established phase of hypertension: SBP decreased from 165 ± 10 to 157 ± 7 mmHg while the excretion of nitric oxide metabolites increased significantly. The glomerulosclerosis index was lower after RIY treatment in both age groups (significant only in young rats 0.29 ± 0.05 vs 0.48 ± 0.04 in the control group; *p* < 0.05). Hence, it seems that peptide RIY exhibits some positive effect on renal morphology. The results obtained suggest that the peptide RIY may be a useful tool in the treatment of hypertension, especially in cases when ACE inhibitors are not effective.

## Introduction

For the past several decades, hypertension has been considered one of the world’s major health threats. WHO reported that about 1.13 billion people worldwide have hypertension and the total direct cost of treatment is estimated at $200 billion by 2030 (DePalma et al., 2018). Hypertension may lead to structural remodeling of blood vessels, heart failure, myocardial infarction, stroke, end-stage renal disease and many other disorders (Kearney et al., 2005; Lackland and Weber, 2015). It is often associated with increased activity of vasoconstrictor angiotensin II (ANG II), the main active peptide in the renin-angiotensin-aldosterone system (RAAS) (Guyton and Hall, 2006). ANG II plays an important role in arterial blood pressure control and it is responsible for the remodeling of the heart and the blood vessel wall. One of the common antihypertensive treatments involves pharmacological inhibition of angiotensin converting enzyme (ACE), which converts angiotensin I (ANG I) to ANG II (Wu et al., 2013). However, this treatment is sometimes not or only partially effective, especially considering miscellaneous etiologies of essential hypertension (Carretero and Oparil, 2000; Park et al., 2010). The phenomenon, often referred to as escape from ACE inhibition or simply “ACE escape”, is right now well recognized, notably pronounced during prolonged treatment and with concomitant disorders such as heart failure (Ennezat et al., 2000). ANG I and renin accumulation due to prolonged ACE inhibition, might in turn overcome the effectiveness of ACEi (ACE *inhibitors*) to suppress RAAS activity (Athyros et al., 2007). Recent research suggest that almost 40% of ANG II is formed through renin-dependent, but ACE-independent processes (Athyros et al., 2007) and other enzymes, such as chymase or elastase 2, can generate ANG II (Okunishi et al., 1987; Givertz, 2001; Kirimura et al., 2005; Morikawa et al., 2005).

Chymase is a serine protease, which has been detected in endothelial and interstitial cells of many organs, such as the heart, blood vessels and kidneys (Okunishi et al., 1987; Guo et al., 2001; Kirimura et al., 2005; Miyake-Ogawa et al., 2005). Moreover, chymase is strictly controlled in normal tissues, but its activation is more pronounced in the states of inflammation and oxidative stress (Urata, 2000; Takai et al., 2004). It can cause the vascular smooth muscles cell apoptosis, also through activation of transforming growth factor β1 and matrix metalloproteinases (MMP-1 and 3), and can lead to degradation of the extracellular matrix. Moreover, ANG II generated by chymase is especially implicated in the remodeling of vasculature (Rosenson, 2009). Chymase is stored in form of inactive pro-chymase in mast cells, wherefrom it can be released into the extracellular matrix in response to certain factors, such as tissue damage (e.g., as a result of inflammation) (Wang et al., 2015).

It is speculated that chymase inhibition might become a new therapeutic strategy to treat some diseases, such as hypertension or diabetes (Wang et al., 2015; Ansary et al., 2018). This might be of particular importance when “ACE escape” phenomenon occurs after chronic treatment with ACEi (Lorenz, 2010).

The aim of the study was to examine if inhibition of ACE-independent pathways of ANG II formation will affect BP, renal and cardiovascular function and morphology of spontaneously hypertensive rats (SHR). This animal model of the disease is considered to mimic human primary hypertension to the best extent, due to its genetic basis and etiology (Doris, 2017; Gawrys et al., 2020).

In our studies we used commercially available chymase inhibitor, chymostatin, and peptides obtained from rapeseed, which were proved previously to possess antihypertensive activity (Marczak et al., 2003; Yamada et al., 2010). Marczak et al. (2003) tested their antihypertensive properties after a single oral administration to SHR, and despite their weak affinity for ACE (1000 times lower than captopril), a significant hypotensive effect was observed. This suggests their inhibitory action toward RAAS enzymes other than ACE. The peptides derived from rapeseed are easy and inexpensive to obtain, which makes them an asset in the combat of hypertension.

Based on our preliminary study in which we tested four different peptides, two with the greatest potential to block chymase were selected for study: RIY (*Arginine-Isoleucine-Tyrosine*, also called rapakinin) and VWIS (*Valine-Tryptophan-Isoleucine-Serine*). Subsequent groups received chymostatin or solvent control (DMSO in saline). The treatments were administered intravenously to young rats in the developmental stage of hypertension (6 weeks old) and to adult SHR in established phase of hypertension (16 weeks old) during 14 days. This is the first study to evaluate the impact of the selected inhibitors on blood pressure, renal function and morphology of the heart and kidney in two stages of hypertension in SHR.

## Materials and Methods

All commercially available solvents and reagents were purchased from Sigma-Aldrich unless otherwise stated.

### Peptide Synthesis

Four peptides: IY (*Isoleucine-Tyrosine*), VW (*Valine-Tryptophan*), RIY (*Arginine-Isoleucine-Tyrosine*; rapakinin) and VWIS (*Valine-Tryptophan-Isoleucine-Serine*) were prepared. All peptides were prepared by solid phase peptide synthesis, using standard Fmoc/tBu methodology. The properly preloaded Wang resins, common Fmoc-protected amino acid building blocks in standard Fmoc-Xxx-OH/TBTU/DIPEA protocol (2eq/2eq/4eq respectively) was used. The desired compounds were produced by deprotection with Reagent B: TFA/phenol/H_2_O/TIPS (88:5:5:2; v/v/v/v). Crude peptides were purified by reverse-phase HPLC (high-performance liquid chromatography), using gradients of H_2_O–ACN–0.1% TFA and the relevant fractions were lyophilized. The peptide purity, estimated by analytical HPLC was more than 95%. The molecular weights were confirmed by HR-ESI-MS (high-resolution electrospray ionization mass spectrometry).

### *In Vitro* Effectiveness of Rapeseed-Derived Peptides to Block Chymase

Chymase digestion of ANG I in the presence of synthetized peptides was performed *in vitro.* The concentration of remaining ANG I was calculated in order to assess the ability of peptides to inhibit chymase.

Human chymase (72 units/mg protein; 259 µg protein/ml, vial volume 187 μl, Sigma) stock solution was prepared by diluting the initial sample of enzyme with Tris*HCl buffer (0.27 mM Tris*HCl, pH 7.8 and 150 mM NaCl) to obtain the final chymase concentration of 1.379 μg/ml. The ANG I (DRVYIHPFHL) stock solution was prepared by dissolving the peptide in the same Tris*HCl buffer (as above) to obtain a final peptide concentration of 1 mM. The peptide basic solutions of each of four peptides (RIY, VWIS, IY, VW) were prepared by dissolving the proper TFA salt of peptide in ANG I stock solution to obtain a final peptide concentration of 5 mM (5-fold excess to ANG I).

The samples of 100 μl of chymase stock solution were temperature-equilibrated at 37 ± 1°C for 15 min before adding 100 μl of angiotensin I stock solution or mixture of proper peptide in angiotensin I stock solution. Reactions (during which ANG I was converted to ANG II through removal of two C-terminal residues) were stopped at the desired times (0, 10, 20, 30, 40, 60, 80 min) by addition of 10 μl of 98% FA. After FA inhibition, reactions were freeze-dried, re-dissolved in 0.5 ml of methanol and analyzed using HPLC-MS system on C-12 column at linear AB gradient of 20–44% for B in 20 min where eluent A was 0.05% trifluoroacetic acid/water and eluent B was 0.05% trifluoroacetic acid/acetonitrile. Positive electrospray (ES+) was used as the ionization method for mass spectrometry.

The inhibitory effect of each peptide on chymase activity was expressed as remaining amount of substrate *vs.* time of digestion and presented as percentage ratio and calculated by the following formula [ANG I_t=X min_/ANG I_t=0 min_]x100% where ANG I_t=X min_ is the peak area of angiotensin I at desired time of digestion, ANG I_t=0 min_ is the peak area of angiotensin I at 0 min of digestion. The results were independently repeated in a second experiment and were used to calculate the half-life time of ANG I in absence or presence of inhibitory peptides.

### *In Vivo* Experiments

#### Experimental Animals

All experimental procedures were approved by the First Ethical Committee for Animal Experimentation in Warsaw, which follows the European Directive 2010/63/EU on the protection of animals used for scientific purposes (permit no: 30/2011, 10/2014, 11/2014, 86/2015, 483/2017). Male, spontaneously hypertensive rats (SHR) were bred in the Mossakowski Medical Research Institute (Polish Academy of Sciences) animal house and were housed in a strictly controlled environment (25 ± 2°C; 12/12 h light–dark cycle, 50–60% humidity). Rats were fed a standard diet (STD, 0.25% Na w/w, SSNIFF GmbH, Soest, Germany) and had free access to drinking water during the whole experiment. SHRs in two phases of hypertension were used: in the early stage (6 weeks old; *n* = 35, mean body weight at the beginning of the experiment: 125 ± 5 g) and in the established phase of hypertension (16 weeks old; *n* = 45; mean body weight: 301 ± 4 g). Rats were randomly divided into the experimental groups. The initial number of animals in each group was usually 7–8, but, depending on the circumstances, the final number might be lower, as stated in the RESULTS chapter, and in the corresponding Figure or Table legend. In some cases, blood pressure measurement was incomplete or impossible, due to technical problems with telemetry probes (clots forming in the transmitters’ cannulas). Only rarely, were some of the animals excluded due to post-operative complications/mortality. All procedures were in accordance with the 3Rs rule to minimize the number of the animals used and reduce their suffering.

### Acute Infusion of Chymostatin to Anesthetized Rats (I)

In acute experiments rats were anesthetized with intraperitoneal (*ip*) sodium thiopental (100 mg/kg; Sandoz GmbH, Kundl, Austria), which provided stable anesthesia for about 4 h. All surgical preparations were already described in detail (Gawrys et al., 2014; Badzynska et al., 2020). Throughout the experiment the temperature of the animal was kept constant (37°C). A cannula was placed in the trachea to ensure a free airway. The jugular vein was cannulated for infusion of fluids and drugs; during surgical preparations 3% bovine albumin in Ringer solution was infused at 10 ml/kg/h. For mean arterial blood pressure (MBP) measurement, a Teflon catheter was placed in the femoral artery (Stoelting Blood Pressure Meter, Wood Dale, IL, United States). After surgical preparations and stabilization of all parameters, a short control period (30 min) was made to obtain baseline for measured parameters. Chymostatin or its solvent (final concentration of DMSO was 0.05%) were administered for 1 h. After cessation of chymostatin infusion, a recovery period (30 min) was made. In the end, the animals were sacrificed by sodium thiopental overdose (*iv*); and vital life signs (blood pressure, breathing) were monitored until death.

### Chronic Experiments (II)

#### Surgical Preparations

Implantation of the telemetry transmitters for BP measurement: Seven days before the start of the chronic experiment all rats were implanted with telemetry probes for blood pressure monitoring (Data Science International, St. Paul, MN, United States). The transmitters (TA11PA-C10 dedicated for young animals or TA11PA-C40 for adult rats, Data Science International, St. Paul, MN, United States), were implanted under aseptic conditions, under sodium pentobarbital anesthesia (Sodium Pentobarbital, dose: 50 mg/kg *ip*, Biowet, Pulawy, Poland). The transmitter’s cannula was implanted into the aorta and transmitter’s body was placed inside the peritoneal cavity and fixed to the abdominal muscle wall. Metacam (0.4 mg/kg BW, Boehringer, Ingelheim, Germany) and Baytril (10 mg/kg BW, Bayer, Leverkusen, Germany) were used as post-operative analgesia and to prevent infection, respectively.

Implantation of cannulas for intravenous administration of selected substances: Four days before the start of the chronic experiment, the femoral vein was cannulated for chronic administration of the substances (Micro-Renathane MRE033, BIOSEB-In Vivo Research Instruments, United States/Canada), and the femoral artery was cannulated for blood sampling (Micro-Renathane Tubing MRE-025 and MRE033, BIOSEB-In Vivo Research Instruments, United States/Canada, respectively in young and adult rats). Both cannulas were filled with 4% Citra-Lock solution (Dirinco B.V, Ketelmeer 1, 5347 JX Oss, Netherlands) to prevent blood clotting and inflammation. After the surgery the post-operative treatment was applied as that for telemetric probe implantation.

#### Protocol and Measurements

Systolic and diastolic blood pressure (SBP and DBP, respectively) and heart rate (HR) were measured in conscious unrestrained rats by telemetry technique, once a week for 24 h. 24 hour observations in metabolic cages (Tecniplast S. p.A. Buguggiate, Italy) were performed weekly. The diuresis, weight of feces, food and water intake were measured; urine and blood samples were collected on day 0, 7th and 14th, and were centrifuged at 1200 g (blood for 15 min and urine for 5 min), then the samples were stored at –80°C until analysis.

On day 0 all animals received saline (0.9% NaCl) and then, for 14 consecutive days, the substance tested. Chymostatin (CH, 2 mg/kg/day) was dissolved in DMSO and then diluted with PBS (final concentration of DMSO was 0.15%). Peptides RIY (7.5 mg/kg/day) and VWIS (12.5 mg/kg/day) were dissolved in saline (0.9% NaCl, S). The substances were administered through the femoral vein during 10 min in the volume of 0.5 ml to young SHR (infusion rate: 3 ml/h) and 1 ml to adult rats (infusion rate: 6 ml/h). Control rats received solvents (either DMSO or saline) in the same volume.

After the chronic experiment rats were anesthetized with sodium thiopental (100 mg/kg, *ip*, Sandoz GmbH, Kundl, Austria) and the heart, kidney and tibia were harvested for morphologic analysis. Additionally, left ventricular hypertrophy was assessed: the hearts were immediately harvested, dried with filter paper, and weighed. Then the left ventricle (LV) was carefully excised and weighed again. Left ventricular hypertrophy was assessed based on the ratio of LV mass to tibia length (LV/tibia).

### Analytical Procedures

Urine volume was determined by gravimetric method. Sodium and potassium concentration in urine and plasma were measured by flame photometry (PFP7/C, Jenway Ltd, Stone, United Kingdom). Urine and plasma osmolality were measured by freezing point depression (Osmomat® 030 M, Gonotec, Berlin, Germany). These parameters were used to determine the excretion rates of total solutes (U_osm_V), sodium (U_Na_V) and potassium (U_K_V). Commercially available ELISA kits were used to measure nitric oxide metabolites (NOx, SunRed, Shanghai) and albumin (Albumin, ICL, Portland) concentration in urine. Excretion rate of NOx (U_NOx_V) and albumin (UAE) were calculated.

### Glomerulosclerosis Index

The kidneys were fixed in 4% buffered formalin, embedded in paraffin and stained with hematoxylin/eosin for morphology evaluation. Glomerulosclerosis/hyalinosis index (GSI) was defined as segmental solidification of the glomerular capillary tuft, segmental collapse, obliteration of capillary lumen and accumulation of hyaline. Fifty glomeruli per section were randomly selected and evaluated using semi-quantitative scoring method as described in detail previously (Maric et al., 2004; Gawrys et al., 2018). Blind group allocation of the investigators was used.

### Statistics

Values are expressed as means ± SEM. The significance of changes was evaluated by multivariate analysis of variance ANOVA with repeated measurements (followed by Duncan’s or Dunnett’s *post hoc* tests). For some parameters one-way ANOVA was used. A value of *p* < 0.05 was considered to be significant. Calculations were made in STATISTICA (version 10.0; StatSoft Inc. Krakow, Poland) or GraphPad Prism (Version 6.07).

## Results

### *In Vitro* Effectiveness of Rapeseed–Derived Peptides to Block Chymase

**FIGURE 1:**
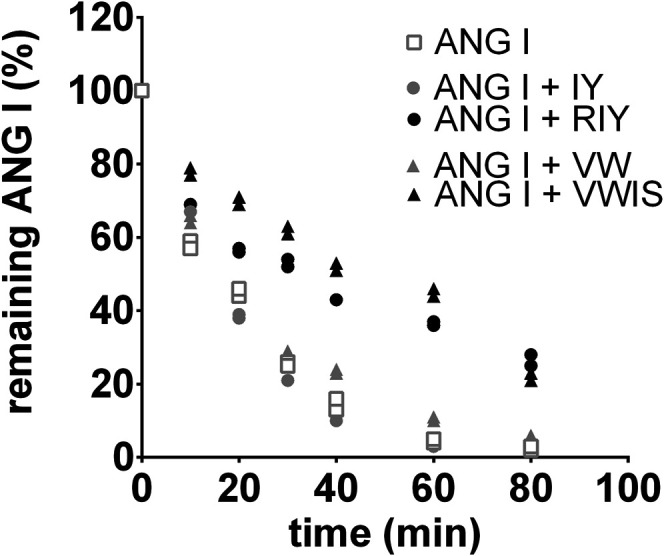
Digestion of angiotensin I (ANG I) in human chymase solution. The peptide was incubated with the enzyme for different time periods in the absence or presence of the inhibitory peptides and the remaining ANG I amounts were determined by reversed-phase high performance liquid chromatography. The results were independently repeated in a second experiment and were used to calculate the half-life time of ANG I in absence or presence of inhibitory peptides (GraphPad Prism Software): T_1/2_ (ANG I) = 16 min; T_1/2_ (ANG I + VW) = 16 min; T_1/2_ (ANG I + IY) = 15 min; T_1/2_ (ANG I + RIY) = 24 min; T_1/2_ (ANG I + VWIS) = 56 min; the mean values were used for the analysis with the SD. *R* ^*2*^ values ranged from 0.9679 to 0.9972. VW: *valine-tryptophan,* IY: *isoleucine-tyrosine,* RIY: *arginine-isoleucine-tyrosine,* VWIS: *valine-tryptophan-isoleucine-serine.*

Out of four tested peptides only RIY and VWIS exhibited blocking activity toward chymase. The conversion of ANG I to ANG II by chymase was visibly slowed down, hence these two peptides were used in subsequent *in vivo* experiments (Figure 1).

### *In Vivo* Experiments

#### Mean Blood Pressure in Acute Experiments

**FIGURE 2:**
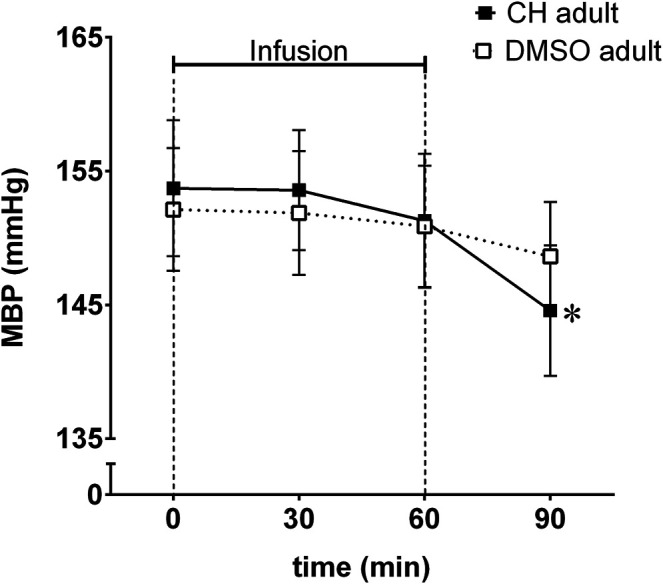
Mean blood pressure (MBP) reordered in acute experiment in anesthetized spontaneously hypertensive rats (SHR) receiving an hour long infusion of chymostatin (CH, 2 mg/kg, *n* = 7) or it’s solvent (0.05% DMSO, *n* = 8); *p ≤ 0.05 vs. baseline value within the same group (multivariate analysis of variance ANOVA with repeated measurements, followed by Duncan’s *post-hoc* test).

Mean blood pressure decreased significantly in anesthetized adult SHR after a single (1 h in duration) intravenous infusion of chymostatin (Figure 2). The decrease in MBP was delayed, observed 30 min after cessation of chymostatin infusion.

#### Chronic Studies

##### Systolic Blood Pressure

**FIGURE 3:**
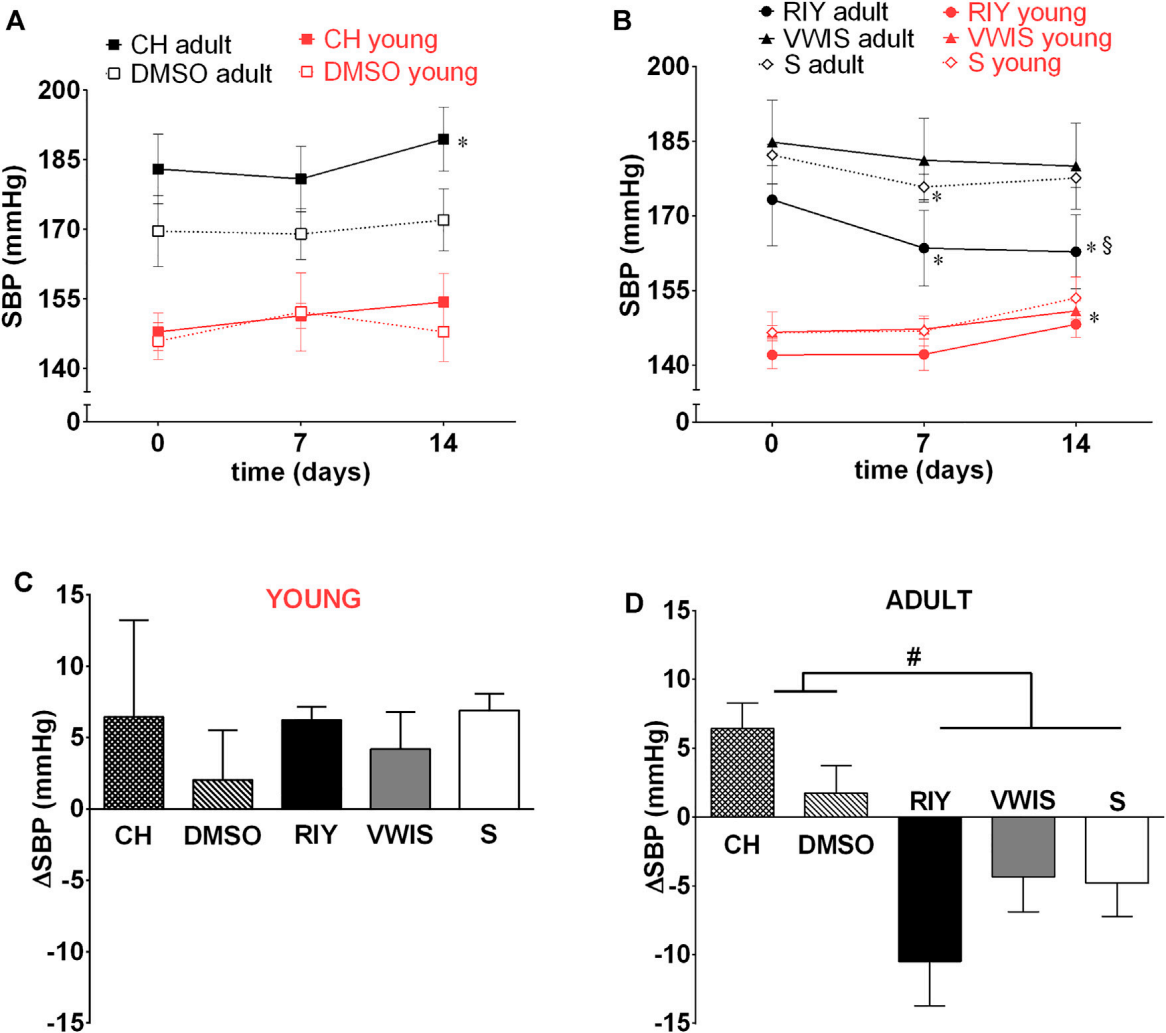
Systolic blood pressure (SBP) measured on 0, 7th and 14th day in young and adult spontaneously hypertensive rats (SHR) receiving intravenous treatment with **(A)** chymostatin (CH, 2 mg/kg/day, young: *n* = 5, adult: *n* = 8) and its solvent (0.15% DMSO, young: *n* = 5, adult: *n* = 5) **(B)** peptides RIY (7.5 mg/kg/day, young: *n* = 6, adult: *n* = 5), VWIS (12.5 mg/kg/day, young: *n* = 5, adult: *n* = 6) and their solvent–saline (S, young: *n* = 5, adult: *n* = 5); **(C)** overall change in SBP (ΔSBP between day 0 and 14) in young and **(D)** adult animals; *p < 0.05 significantly different *vs.* baseline value on day 0 within the same group; § value in RIY *vs.* CH group on day 14th (*p* < 0.05; multivariate analysis of variance ANOVA with repeated measurements, followed by Duncan’s *post-hoc* test); #*p* < 0.05 significantly different values between indicated groups (one-way ANOVA, followed by Duncan’s *post-hoc* test).

Neither of the inhibitors applied (chymostatin and the both rapeseed-derived peptides) affected blood pressure in young animals (Figure 3). In all tested groups slight increase in SBP was observed corresponding to the typical progression of hypertension in SHR.

After chronic intravenous infusion of chymostatin no sustained decrease in arterial blood pressure was observed in adult SHR. Similarly as with acute experiments, only transient BP reduction was recorded during the first 7 h post-infusion. Interestingly, after the first week SBP started to increase in chymostatin-treated group and in the end it was significantly higher than at the beginning of the experiment.

Of all the substances tested, only RIY exhibited significant antihypertensive activity (Figure 3B): after 7 days of treatment a significant decrease in SBP was recorded. The hypotensive effect was stable and lasted till the end of the experiment. The values of SBP in RIY-treated group were significantly lower in comparison to the chymostatin group. Intravenous VWIS did not exhibit any antihypertensive activity.

There were no differences in the heart rate between the experimental groups (Supplementary Figure S1).

##### Metabolic, Plasma and Urine Parameters

All animals were in good health and displayed normal physical activity; the treatments applied did not affect basic metabolic, plasma and urine parameters. We observed some minor even though significant fluctuations during the experiment, evidently physiologically meaningless. All the parameters were within the normal range for SHR (see Supplementary Tables S1–4). After RIY treatment, only in adult rats with established hypertension, water intake and diuresis increased slightly, however, there were no differences between groups (Supplementary Table S2).

##### Excretion of Nitric Oxide Metabolites and Albumin

**FIGURE 4:**
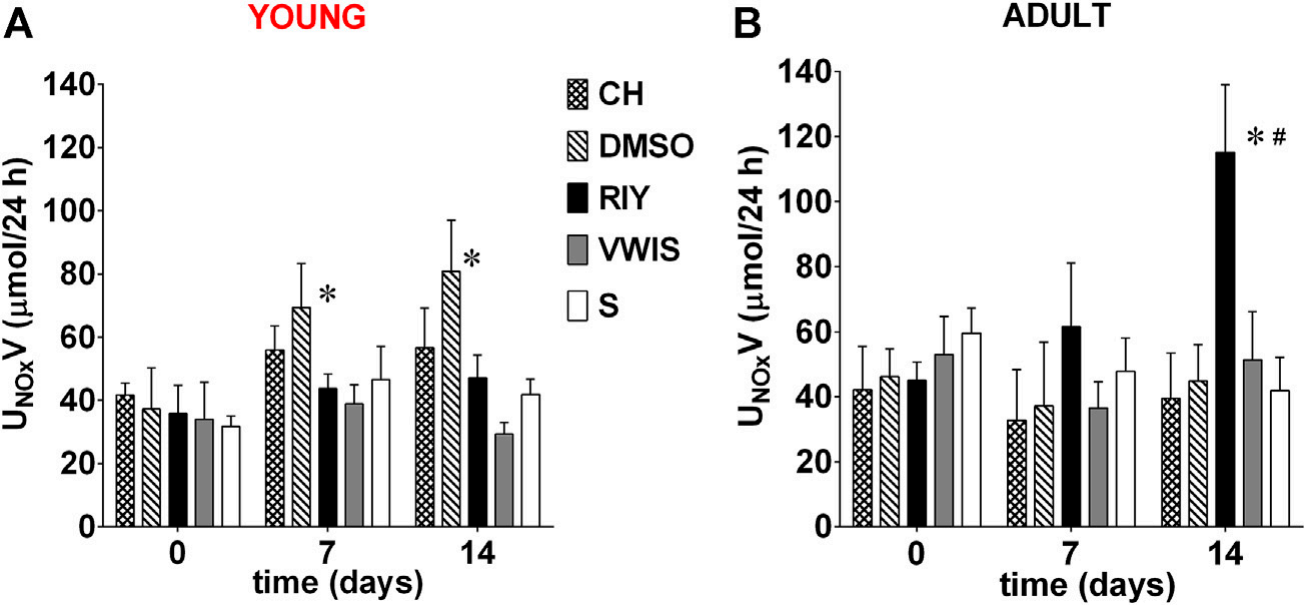
Excretion of nitric oxide metabolites (U_NOx_V) measured on 0, 7th and 14th day in **(A)** young and **(B)** adult spontaneously hypertensive rats (SHR) receiving intravenous treatment with chymostatin (CH, 2 mg/kg/day, young: *n* = 6, adult: *n* = 8), its solvent (0.15% DMSO, young: *n* = 6, adult: *n* = 5); peptides RIY (7.5 mg/kg/day, young: *n* = 5, adult: *n* = 5), VWIS (12.5 mg/kg/day, young: *n* = 5, adult: *n* = 5) and their solvent–saline (S, young: *n* = 5, adult: *n* = 4); *p < 0.05 significantly different *vs.* baseline value on day 0 within each group; # significantly different for RIY-treated group *vs.* values in all other groups on 14 days (*p* < 0.05; multivariate analysis of variance ANOVA with repeated measurements, followed by Dunnet’s *post-hoc* test).

The excretion rate of nitric oxide metabolites significantly increased in young rats which received chymostatin solvent (DMSO). In adult rats only, RIY-treated group showed a significant increase in nitric oxide excretion (Figure 4). The value was statistically higher than in all other groups after two weeks of treatment (day 0: 0.05 ± 0.01 vs. day 14: 0.12 ± 0.02*, *p* < 0.05).

Urinary excretion of albumin (UAE) in young animals significantly increased in all experimental groups, except for the control group receiving saline (S). However, in this group the baseline level of albumin was distinctly higher than in other groups, hence no significant increase was observed (Table 1). Moreover, there were no substantial differences in UAE between groups, irrespective of the chymase inhibitor applied. No significant changes in albumin excretion was observed in adult animals (Table 1). In general, UAE values were relatively low in all groups: much higher values are considered a symptom of abnormal albuminuria in rats. It is increasingly accepted that albumin excretion is not a good marker of kidney damage in SHR (however, it is still routinely determined), at least not one to indicate an early renal injury, what was repeatedly shown in previous studies, including our own (Garrett et al., 2006; Inoue et al., 2013; Gawrys et al., 2018).

**TABLE 1:** Urinary albumin excretion (UAE) measured on 0, 7th and 14th in young and adult spontaneously hypertensive rats (SHR) receiving intravenous treatment.

mg/24h	CH	DMSO	S	RIY	VWIS
Young SHR					
Day 0	0.03 ± 0.01	0.04 ± 0.01	0.07 ± 0.02	0.03 ± 0.01	0.06 ± 0.02
Day 7	0.07 ± 0.01	0.06 ± 0.01	0.07 ± 0.02	0.09 ± 0.01*	0.07 ± 0.02
Day 14	0.13 ± 0.03*	0.11 ± 0.02*	0.08 ± 0.02	0.14 ± 0.02*	0.11 ± 0.02*
Adult SHR					
Day 0	0.59 ± 0.33	0.53 ± 0.33	0.45 ± 0.22	0.51 ± 0.27	0.53 ± 0.23
Day 7	0.41 ± 0.04	0.41 ± 0.12	0.48 ± 0.26	0.58 ± 0.19	0.97 ± 0.31
Day 14	0.84 ± 0.32	0.64 ± 0.38	0.42 ± 0.18	0.72 ± 0.41	0.91 ± 0.24

CH–chymostatin (2 mg/kg/day; n = 5); DMSO–solvent (0.15%; n = 4–5); peptides RIY (7.5 mg/kg/day; n = 5); VWIS (12.5 mg/kg/day; n = 5); S–saline (n = 5). *p < 0.05 *vs.* day 0 (multivariate analysis of variance ANOVA with repeated measurements, followed by Duncan’s *post-hoc* test).

##### The Glomerulosclerosis Index and Left Ventricular Hypertrophy

Of all chymase inhibitors tested, only RIY significantly decreased GSI in young animals (in comparison to the saline group; Table 2). In adult rats with established hypertension GSI tended to be lower in all treated groups (those receiving chymostatin and RIY and VWIS) in comparison to solvent treatment (DMSO or saline). However, in chymostatin group only the change reached statistical significance (*vs.* DMSO). For RIY and VWIS, GSI only tended to change (compared to saline group; *p = 0.08* for both groups *vs.* S).

**TABLE 2:** The glomerulosclerosis index (GSI) and left ventricular hypertrophy (LV/tibia).

	CH	DMSO	S	RIY	VWIS
Young SHR					
GSI	0.51 ± 0.07	0.36 ± 0.06	0.48 ± 0.04	0.29 ± 0.05*	0.47 ± 0.11
LV/tibia (mg/mm)	16.7 ± 0.9	15.2 ± 1.1	15.7 ± 0.6	16.2 ± 1.0	15.2 ± 0.8
Adult SHR					
GSI	0.50 ± 0.07#	0.75 ± 0.05	1.04 ± 0.23	0.56 ± 0.09	0.55 ± 0.09
LV/tibia (mg/mm)	20.7 ± 2.6	22.6 ± 2.3	20.8 ± 1.3	22.2 ± 1.1	21.5 ± 1.0

CH–chymostatin (2 mg/kg/day); DMSO–solvent (0.15%); peptides RIY (7.5 mg/kg/day); VWIS (12.5 mg/kg/day); S–saline (n = 5 in each group). *p < 0.05 vs. S. # p < 0.05 vs. DMSO (one-way ANOVA followed by Duncan’s post-hoc test).

The ratio of the left ventricular mass to tibia length (LV/tibia) did not differ between groups in either phase of hypertension (early or established).

## Discussion

The aim of the study was to investigate the involvement of ACE-independent pathway of ANG II production in blood pressure regulation and to evaluate the clinical usefulness of novel rapeseed-derived chymase inhibitors for antihypertensive therapy. The most significant finding of the study is that peptide RIY appeared to have beneficial influence on blood pressure level, cardiovascular and renal function of adult SHR. If confirmed by further research, this finding could find application in the clinic as a part of antihypertensive therapy and/or could be used as functional food.

### Chymostatin

Analysis of preliminary acute experiments with intravenous administration of a single dose of chymostatin to anesthetized rats, showed some antihypertensive properties. The observed decrease in blood pressure was significant but delayed, observed after cessation of chymostatin infusion, which suggested some role in blood pressure regulation in adult SHR. These results prompted us to study the effects of chymostatin after chronic administration to conscious rats, to evaluate possible prolonged inhibition of chymase on blood pressure.

Two-week treatment with chymostatin did not lower blood pressure in SHR. Surprisingly, this commercially available chymase inhibitor had an opposite effect, and a significant increase in SBP was observed.

Kirimura et al. (2005), studied NK3201, another chymase inhibitor. The authors found that a single oral dose of NK3201 did not lower blood pressure in adult SHR and concluded that chymase pathway of ANG II formation does not play an important role in this rat model of hypertension. However, the study by Kirimura et al. (2005), in addition to the obvious difference in the protocol (single oral dose *vs.* our chronic intravenous infusion) has another limitation: the tail cuff method was used to measure blood pressure. This method is now considered far from ideal and does not allow to record subtle BP changes (Wilde et al., 2017). Moreover, spontaneously hypertensive rats are susceptible to stress, *e.g.,* that due to prolonged immobilization during the tail cuff measurement, which might contribute to the hypertension development (Yamori, 1977; Henry et al., 1993).

Even though we obtained the results similar as Kirimura et al. (2005), we do not dismiss the importance of chymase in ANG II formation. We suggest that chronical inhibition of local/tissue RAAS results in activation of the main pathway of ANG II production, through systemic ACE. It is known that chymostatin does not block ACE (Petrie et al., 2001), hence more of endogenously generated ANG I is available for ACE. It was previously suggested that inhibition of ACE alone does not suffice to suppress RAAS (Petrie et al., 2001), which provides a rationale to employ chymase inhibitors to treat hypertension. It seems that blocking both pathways, at least partially, should be considered as a new therapeutic option.

Surprisingly, administration of DMSO in young animals resulted in a substantial increase in the excretion rate of nitric oxide metabolites. This somewhat confounding finding calls for special attention. We believe that young rats are more susceptible to toxic effects of DMSO, yet since they are still normotensive with healthy endothelium, the observed increase might be caused by NOS stimulation as a counteraction to vasoconstriction due to DMSO. Such mobilization seems more likely in young animals, because of their endothelium is not yet damaged by hypertension and in adult rats this mechanisms is probably severely impaired. Moreover, it seems that susceptibility to DMSO toxicity of adult rats is lower. Assuredly, this speculation requires more in-depth studies in the future.

### Rapeseed-Derived Peptides

Previous research by our collaborators demonstrated that rapeseed-derived peptides possess some antihypertensive potential, despite only weak affinity toward ACE (Marczak et al., 2003; Yamada et al., 2010; Yamada et al., 2011). Current *in vitro* research confirmed that they also inhibit chymase, therefore we tested them further in prolonged, chronic studies in conscious SHR. The most promising effects were observed after RIY administration (rapakinin). It significantly reduced blood pressure, and increased nitric oxide (NO) metabolite excretion almost 3-fold. Also the glomerulosclerosis index (GSI) was lower, suggesting that RIY alleviated renal impairment to some extent. It is worth emphasizing that the beneficial action of RIY was observed in adult rats in established phase of the disease, with already developed renal and cardiovascular damage, a condition which represents a greater challenge than prevention of the disease in young subjects.

Based on our results it seems that chymase does not play any significant role in the developmental phase of hypertension, since no blood pressure or any other change was observed after chymase inhibition (similarly after chymostatin and rapeseed-derived peptides). This is somewhat surprising, considering previous reports suggesting the important role of mast cells, the main source of chymase, in tissue fibrosis (Kotov et al., 2020). Possibly, mast cells-related tissue remodeling might be more pronounced in more advanced hypertensive heart disease. Chymase-related tissue remodeling probably occurs also in the kidneys and may be a strong contributor to hypertension in older SHR, however this needs to be confirmed in further research.

In view of the above considerations, we suggest that the reason for the significantly higher effectiveness of rapakinin (when compared to chymostatin) is its ability to block both systemic and local pathway of ANG II production. Previous study by Marczak et al. (2003) also demonstrated that RIY is more stable in the presence of ACE, i.e., is not converted to less active dipeptide IY in such quantity as VWIS to VW. The study by Yamada *et al.* with isolated mesenteric arteries of SHR, demonstrated that RIY relaxes the vessels *via* an endothelium-dependent mechanism (Yamada et al., 2010). The authors claim that the vasoactive action of RIY is not mediated through nitric oxide and bradykinin. In the present study we observed significantly higher excretion of NO metabolites in rats receiving RIY (in which significant reduction in blood pressure was recorded). This observation somehow contradicts the NO-independent pathway suggested by the aforementioned authors*.* However, it should be noted that experiments on isolated tissues differ significantly from functional studies performed on whole conscious animals, in which the observed effect is a result of interaction of many complex regulatory systems.

Another important element in the complex nature of RAAS function is the existence of other substrates which can be converted to angiotensin II, such as angiotensin (1–12). It is suggested that ANG (1–12) is a superior substrate for chymase compared to ANG I (Ahmad et al., 2016). Additionally, it has been shown that chymase, not ACE, is the main enzyme generating ANG II from ANG (1–12) in the heart (Ahmad et al., 2011; Ahmad et al., 2013; Nagata et al., 2015; Baghel et al., 2016; De Mello et al., 2016). Recently, the role of intracellular RAAS is increasingly recognized and the discovery of non-canonical, intracellular ANG II production might explain the limited effectiveness of RAAS inhibitors (both *ACE inhibitors* and ARBs i.e., *angiotensin II receptor blockers*), because of their inability to reach intracellular sites and to inhibit chymase-dependent production of ANG II from other substrates, like ANG (1–12) (Ferrario and Mullick, 2017). One of the new suggested strategies to improve antihypertensive therapy is to develop therapeutic agents, such as chymase by RIY, which will be able to penetrate renal and cardiac tissues and inhibit intracellular components of the RAAS (Ferrario and Mullick, 2017).

Lately, protein therapy with so called cell-penetrating peptides (CPPs) is gaining more importance (Milletti, 2012; Ramsey and Flynn, 2015; Patel et al., 2019). CPPs are small molecules which possess the ability to cross the biological membranes and deliver therapeutics into the cells, usually rich in arginine. This amino-acid is involved in the mechanism of cell internalization by CPPs, due to its ability to disrupt and cross phospholipid bi-layers (Takeuchi and Futaki, 2016). Interestingly, RIY is a small tripeptide containing arginine, which could potentially augment its ability to pass across cell membrane and act inside the cells. It is possible that the significant effectiveness of rapakinin is a result of not only blocking two main pathways of ANG II synthesis (chymase and ACE), but also of its ability to penetrate the cell membrane and inhibiting intracellular synthesis of ANG II from ANG (1–12). However, more complex membrane permeability studies are necessary to prove this hypothesis.

The second of the tested peptides, VWIS, did not exhibit any antihypertensive activity. A previous study by Marczak et al. (2003) demonstrated that of the two peptides, VWIS exhibited higher affinity to block ACE than did RIY, however, it was also showed that in the presence of ACE it transformed rapidly to dipeptide VW, which in the present experiments failed to block chymase (see Figure 1). The aforementioned authors conducted also *in vivo* studies in which they measured blood pressure during 8 h following a single oral administration of selected peptides (RIY, VWIS and VW) to SHR. Interestingly, they observed transient (lasting 2 h) significant blood pressure reduction after each of the peptides (Marczak et al., 2003). Therefore we expected that prolonged administration of the peptides (over two weeks) will result in maintained blood pressure reduction in SHR. This was so only in the case of RIY. Possibly, the reason for the lack of antihypertensive activity of VWIS is its transformation to less active VW, which inhibits ACE only weakly and does not block chymase.

### Summary, Conclusion and Limitations

In adult rats the highest antihypertensive activity was observed after treatment with RIY, which might be a result of a few factors. First, it is probable that rapakinin affects both the chymase and ACE pathway of ANG II synthesis. Moreover, RIY contains arginine, which could augment the cell membrane penetration, act inside the cells and inhibit the generation of ANG II from ANG (1–12). Such complex action could account for the positive effects of RIY treatment: a decrease in BP, lowering of GSI and an increase in the excretion of nitric oxide metabolites.

It should be noted that the observed decrease in blood pressure after rapakinin was significant, however not as high as after treatment with the currently used drugs, such as ACEi. More extensive studies are necessary to fully evaluate the clinical value of RIY: prolongation of the treatment and/or elevation of the dose might be useful. Additionally, the efficacy of oral administration of the selected peptides should be examined. Previous studies demonstrated a positive but transient antihypertensive activity of a single oral dose of RIY in SHR (Marczak et al., 2003), therefore tests with chronic intra-gastric administration of the peptides are needed.

In summary, it appears that the peptide RIY might be useful in the treatment of hypertension, especially in the cases resistant to other therapy. Notably, rapeseed-derived peptides are easy and cheap to obtain and therefore might find application as an ingredient of functional food.

## Data Availability

The original contributions presented in the study are included in the article/Supplementary Material, further inquiries can be directed to the corresponding author.
