# Supplementary material for: Chymase Dependent Pathway of Angiotensin II Generation and Rapeseed Derived Peptides for Antihypertensive Treatment of Spontaneously Hypertensive Rats
Source: Front Pharmacol. 2021 May 17;12:658805. doi: 10.3389/fphar.2021.658805 (PMC8165439; doi:10.3389/fphar.2021.658805)
Supplement: Supplementary file 1 [file DataSheet1.docx]

Supplementary Material

Title: “Chymase dependent pathway of angiotensin II generation and rapeseed derived peptides for antihypertensive treatment of spontaneously hypertensive rats”

*Iwona Baranowska et al.*


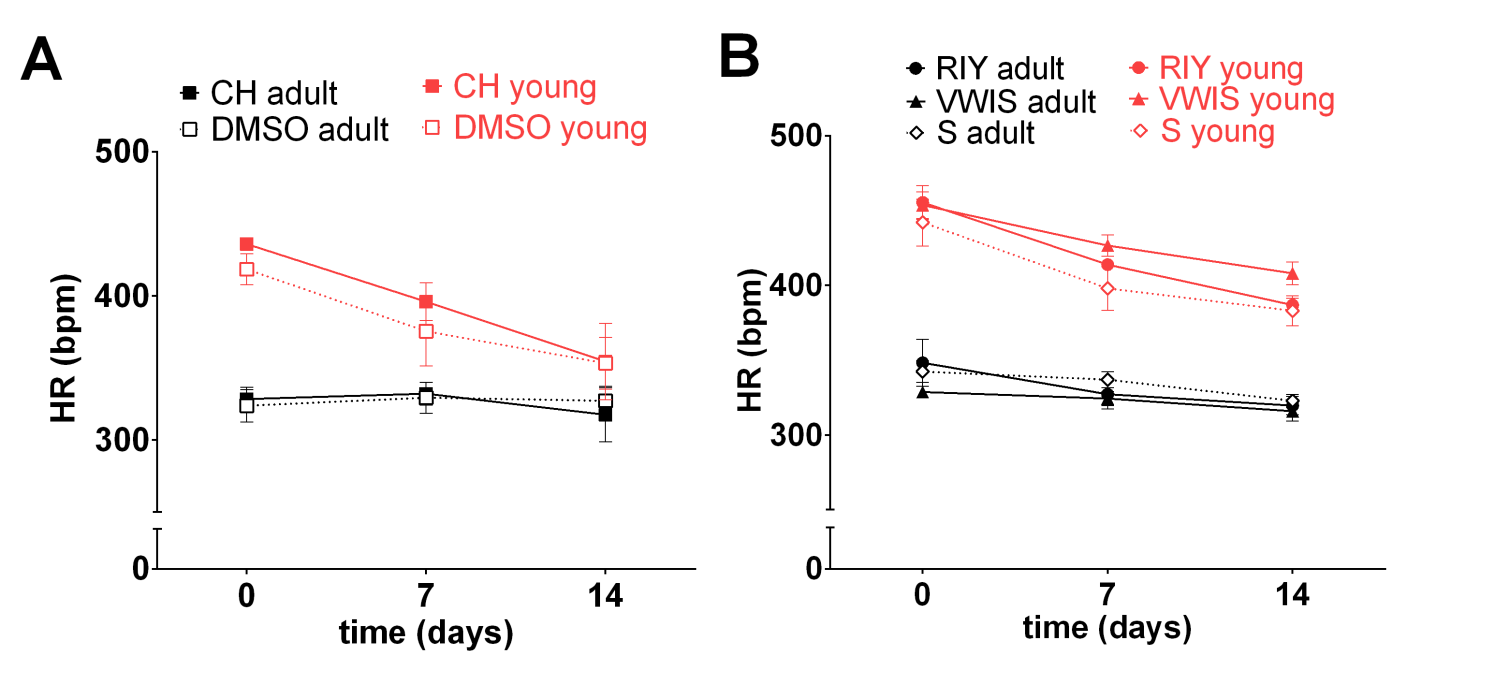


**Supplementary Figure S1**

Heart rate (HR) measured on 0, 7^th^ and 14^th^ day in young and adult spontaneously hypertensive rats (SHR) receiving intravenous treatment with (A) chymostatin (CH, 2 mg/kg/day, young: n=5, adult: n=8) and its solvent (0.15 % DMSO, young: n=5, adult: n=5); (B) peptides RIY (7.5 mg/kg/day, young: n=6, adult: n=5), VWIS (12.5 mg/kg/day, young: n=5, adult: n=6) and their solvent – saline (S, young: n=5, adult: n=5);

Supplementary Table S1

Metabolic parameters measured on 0, 7^th^ and 14^th^ day in young spontaneously hypertensive rats (SHR-6) receiving intravenous treatment with selected substances.

| parameters | Day | Experimental groups | | | | |
| --- | --- | --- | --- | --- | --- | --- |
|  |  | **SHR-6 CH** | **SHR-6 DMSO** | **SHR-6 S** | **SHR-6 RIY** | **SHR-6 VWIS** |
| Body weight (g) | 0 | 131±10 | 126±4 | 124±12 | 112±7 | 110±11 |
|  | 7 | 159±5 | 163±7 | 161±13 | 149±8 | 145±13 |
|  | 14 | 196±4 | 203±4 | 192±11 | 185±7 | 174±13 |
| Food intake (g/24 h) | 0 | 15±1 | 15±1 | 17±1 | 16±1 | 16±1 |
|  | 7 | 18±2 | 20±1 | 19±1 | 19±1 | 19±1 |
|  | 14 | 20±2 | 21±1 | 22±2 | 21±1 | 20±1 |
| Water intake  (g/24 h) | 0 | 23±2 | 27±2 | 25±2 | 23±2 | 22±2 |
|  | 7 | 29±5 | 29±2 | 24±3 | 26±4 | 32±7 |
|  | 14 | 32±3* | 36±2*# | 30±2* | 33±3* | 30±2* |
| Faeces (g/24 h) | 0 | 7±1 | 8±1 | 11±1 | 11±1 | 10±1 |
|  | 7 | 11±1 | 11±1 | 11±2 | 16±1 | 13±1 |
|  | 14 | 12±1 | 12±1 | 15±1 | 15±1 | 14±1 |
| Diuresis (µl/min) | 0 | 6.1±0.5 | 5.3±0.8 | 5.7±1.1 | 5.3±0.8 | 6.3±0.8 |
|  | 7 | 9.9±1.4* | 8.6±0.6* | 7.8±2.1 | 9.0±1.3 | 7.2±0.7 |
|  | 14 | 10.0±0.9* | 13.2±1.4* | 9.0±0.9* | 9.4±1.2 | 8.5±0.8*# |

CH- chymostatin (2 mg/kg/day, n=8-10); 0.15 % DMSO - solvent (n=8); peptides RIY (7.5 mg/kg/day, n=5); VWIS (12.5 mg/kg/day, n=5-6); S – saline (n=4-6); *P<0.05 significantly different *versus* baseline value on day 0 within the same group; #significantly different *versus* value obtained on day 7 within the same group (multivariate analysis of variance ANOVA with repeated measurements, followed by Duncan’s *post-hoc* test).

**Supplementary Table S2**

Metabolic parameters measured on 0, 7^th^ and 14^th^ day in adult spontaneously hypertensive rats (SHR-16) receiving intravenous treatment with selected substances.

| parameters | Day | Experimental groups | | | | |
| --- | --- | --- | --- | --- | --- | --- |
|  |  | **SHR-16 CH** | **SHR-16 DMSO** | **SHR-16 S** | **SHR-16 RIY** | **SHR-16 VWIS** |
| Body weight (g) | 0 | 271±16 | 290±11 | 298±8 | 299±12 | 304±8 |
|  | 7 | 285±17 | 304±11 | 319±10 | 309±9 | 312±11 |
|  | 14 | 298±19 | 313±13 | 332±11 | 326±8 | 329±11 |
| Food intake (g/24 h) | 0 | 21±1 | 17±1 | 21±2 | 20±2 | 20±1 |
|  | 7 | 17±1 | 17±1 | 23±4 | 22±2 | 22±1 |
|  | 14 | 20±2 | 18±1 | 21±2 | 21±1 | 19±1 |
| Water intake  (g/24 h) | 0 | 29±3 | 28±4 | 35±7 | 28±2 | 30±1 |
|  | 7 | 29±3 | 33±3 | 32±4 | 35±2* | 34±1* |
|  | 14 | 29±4 | 31±3 | 30±3 | 37±3* | 32±2 |
| Faeces (g/24 h) | 0 | 9±1 | 7±1 | 11±1 | 10±1 | 11±1 |
|  | 7 | 8±1 | 8±2 | 10±1 | 11±1 | 11±2 |
|  | 14 | 9±1 | 8±1 | 9±1 | 13±1 | 11±1 |
| Diuresis (µl/min) | 0 | 6.9±1.9 | 6.3±1.9 | 10.0±1.0 | 6.4±0.2 | 7.1±1.4 |
|  | 7 | 7.4±2.1 | 5.6±2.0 | 9.6±0.7 | 8.5±1.8 | 7.0±1.3 |
|  | 14 | 8.1±2.9 | 7.7±2.8 | 9.5±0.4 | 10.4±1.4* | 8.0±1.8 |

CH- chymostatin (2 mg/kg/day, n=8); 0.15 % DMSO - solvent (n=5); peptides RIY (7.5 mg/kg/day, n=5-6); VWIS (12.5 mg/kg/day, n=5-6); S – saline (n=5); *P<0.05 significantly different *versus* baseline value on day 0 within the same group (multivariate analysis of variance ANOVA with repeated measurements, followed by Duncan’s *post-hoc* test).

**Supplementary Table S3**

**Plasma and urine parameters measured in samples collected on 0, 7^th^ and 14^th^ day in young spontaneously hypertensive rats (SHR-6) receiving intravenous treatment with selected substances.**

| parameters | Day | Experimental groups | | | | |
| --- | --- | --- | --- | --- | --- | --- |
|  |  | **SHR-6 CH** | **SHR-6 DMSO** | **SHR-6 S** | **SHR-6 RIY** | **SHR-6 VWIS** |
| Plasma osmolality  (mOsm/l) | **0** | 303±3 | 309±12 | 303±2 | 298±1 | 295±2 |
|  | **7** | 297±3* | 298±3 | 300±2 | 298±2 | 297±1 |
|  | **14** | 295±6 | 302±1 | 301±2 | 290±2 | 293±3 |
| Plasma Na+ concentration (mmol/l) | **0** | 125.8±2.1 | 125.3±1.7 | 124.5±3.9 | 130.6±4,8 | 131.8±3.0 |
|  | **7** | 126.0±2.3 | 129.0±3.0 | 124.3±6,1 | 129.6±4.0 | 133.40±5.6 |
|  | **14** | 126.0±1.6 | 126.1±1.8 | 122.3±5.0 | 124.2±4.3 | 131.4±4.5 |
| Plasma K+ concentration (mmol/l) | **0** | 4.34±0.16 | 4.45±0.43 | 4.21±0.26 | 4.43±0.15 | 4.42±0.20 |
|  | **7** | 4.61±0.12* | 4.65±0.33 | 3.72±0.25 | 4.54±0.11 | 4.45±0.16 |
|  | **14** | 3.98±0.13* | 4.77±0.20 | 3.81±0.12 | 4.39±0.18 | 4.33±0.14 |
| Total solute excretion  (µOsm/min) | **0** | 2.94±0.68 | 5.65±0.64 | 6.90±1.28 | 4.84±1.13 | 5.48±1.40 |
|  | **7** | 6.05±1.16* | 5.86±1.82 | 9.35±1.54 | 4.85±1.11 | 7.11±0.95 |
|  | **14** | 7.82±0.47* | 6.97±2.73 | 8.66±1.25 | 6.44±2.35 | 7.16±0.75 |
| Sodium excretion  (µmol/min) | **0** | 0.51±0.09 | 0.54±0.64 | 0.66±0.10 | 0.63±0.24 | 0.69±0.13 |
|  | **7** | 0.90±0.12* | 1.13±0.19* | 0.88±0.22 | 0.64±0.16 | 0.84±0.13 |
|  | **14** | 0.93±0.47* | 1.22±0.1* | 0.90±0.16 | 0.68±0.19 | 0.63±0.08 |
| Potassium excretion  (µmol/min) | **0** | 0.48±0.08 | 0.56±0.17 | 0.68±0.16 | 0.72±0.24 | 0.73±0.20 |
|  | **7** | 0.95±0.12* | 1.20±0.16* | 0.90±0.38 | 0.65±0.09 | 1.75±0.15 |
|  | **14** | 1.01±0.60* | 1.39±0.18* | 0.58±0.14 | 0.71±0.40 | 0.72±0.11 |

CH- chymostatin (2 mg/kg/day, n=8-10); 0.15 % DMSO - solvent (n=8); peptides RIY (7.5 mg/kg/day, n=5); VWIS (12.5 mg/kg/day, n=5-6); S – saline (n=4-6); *P<0.05 significantly different *versus* baseline value on day 0 within the same group; #significantly different *versus* value obtained on day 7 within the same group (multivariate analysis of variance ANOVA with repeated measurements, followed by Duncan’s *post-hoc* test)

**Supplementary Table S4**

**Plasma and urine parameters measured in samples collected on 0, 7^th^ and 14^th^ day in adult spontaneously hypertensive rats (SHR-16) receiving intravenous treatment with selected substances.**

| parameters | Day | Experimental groups | | | | |
| --- | --- | --- | --- | --- | --- | --- |
|  |  | **SHR-16 CH** | **SHR-16 DMSO** | **SHR-16 S** | **SHR-16 RIY** | **SHR-16 VWIS** |
| Plasma osmolality  (mOsm/l) | **0** | 307±2 | 305±2 | 293±6 | 306±4 | 313±1 |
|  | **7** | 299±2* | 294±1 | 294±7 | 304±1 | 308±1* |
|  | **14** | 294±5 | 289±4* | 308±6* | 296±3* | 302±2*# |
| Plasma Na+ concentration (mmol/l) | **0** | 134.6±2.6 | 132.6±2.4 | 126.2±1.8 | 121.8±3.4 | 133.0±0.8 |
|  | **7** | 130.5±2.3* | 129.4±3.1 | 124.1±2.9 | 128.8±2.4* | 130.2±4.5 |
|  | **14** | 126.1±1.0 * | 129.0±2.4 | 124.9±2.1 | 131.3±2.0 | 135.8±4.7# |
| Plasma K+ concentration (mmol/l) | **0** | 4.39±0.27 | 4.63±0.19 | 4.55±0.20 | 3.67±0.20 | 4.20±0.18 |
|  | **7** | 4.21±0.10 | 4.71±0.16 | 4.39±0.15 | 3.81±0.14 | 3.78±0.31 |
|  | **14** | 3.95±0.20 | 4.06±0.30 | 4.00±0.30 | 3.23±0.27 | 3.96±0.06 |
| Total solute excretion  (µOsm/min) | **0** | 7.05±0.90 | 8.02±0.24 | 9.98±1.84 | 10.38±1.82 | 7.52±1.54 |
|  | **7** | 7.11±1.34 | 7.49±0.97 | 13.97±2.23 | 10.41±1.87 | 9.90±2.21 |
|  | **14** | 7.04±1.15 | 9.25±0.45 | 11.98±2.28* | 9.67±1.41 | 10.42±2.06 |
| Sodium excretion  (µmol/min) | **0** | 1.01±0.13 | 0.98±0.18 | 1.29±0.16 | 1.03±0.08 | 0.90±0.18 |
|  | **7** | 0.77±0.23 | 0.96±0.29 | 1.17±0.14 | 0.68±0.16 | 1.02±0.19 |
|  | **14** | 1.11±0.33 | 1.66±0.20 | 0.94±0.14* | 0.86±0.27 | 0.81±0.16 |
| Potassium excretion  (µmol/min) | **0** | 1.28±0.19 | 1.42±0.25 | 2.13±0.21 | 0.93±0.09 | 0.97±0.21 |
|  | **7** | 1.24±0.25 | 1.18±0.08 | 2.25±0.28 | 0.85±0.22 | 1.26±0.27 |
|  | **14** | 1.71±0.15 | 0.85±0.39 | 2.42±0.15 | 1.12±0.18 | 0.95±0.21 |

CH – chymostatin (2 mg/kg/day, n=5-8); 0.15 % DMSO - solvent (n=5); peptides RIY (7.5 mg/kg/day, n=5-6); VWIS (12.5 mg/kg/day, n=5-6); S – saline (n=5); *P<0.05 significantly different *versus* baseline value on day 0 within the same group; #significantly different *versus* value obtained on day 7 within the same group (multivariate analysis of variance ANOVA with repeated measurements, followed by Duncan’s *post-hoc* test)
